# Small-Molecule Disruptors of the Interaction between Calcium- and Integrin-Binding Protein 1 and Integrin α_IIb_β_3_ as Novel Antiplatelet Agents

**DOI:** 10.1021/acsptsci.4c00026

**Published:** 2024-05-29

**Authors:** Kalyan Golla, Adam Yasgar, Voddarahally N. Manjuprasanna, Meghna U. Naik, Bolormaa Baljinnyam, Alexey V. Zakharov, Sankalp Jain, Ganesha Rai, Ajit Jadhav, Anton Simeonov, Ulhas P. Naik

**Affiliations:** † Cardeza Center for Hemostasis, Thrombosis, and Vascular Biology, Cardeza Foundation for Hematologic Research, Department of Medicine, Thomas Jefferson University, Philadelphia, Pennsylvania 19107, United States; ‡ National Center for Advancing Translational Sciences, National Institutes of Health, Rockville, Maryland 20850, United States

**Keywords:** calcium- and integrin-binding protein 1 (CIB1), fluorescence polarization (FP), inhibitors, high-throughput screening (HTS), classical quantitative structure−activity relationship (QSAR), protein−protein interactions (PPIs)

## Abstract

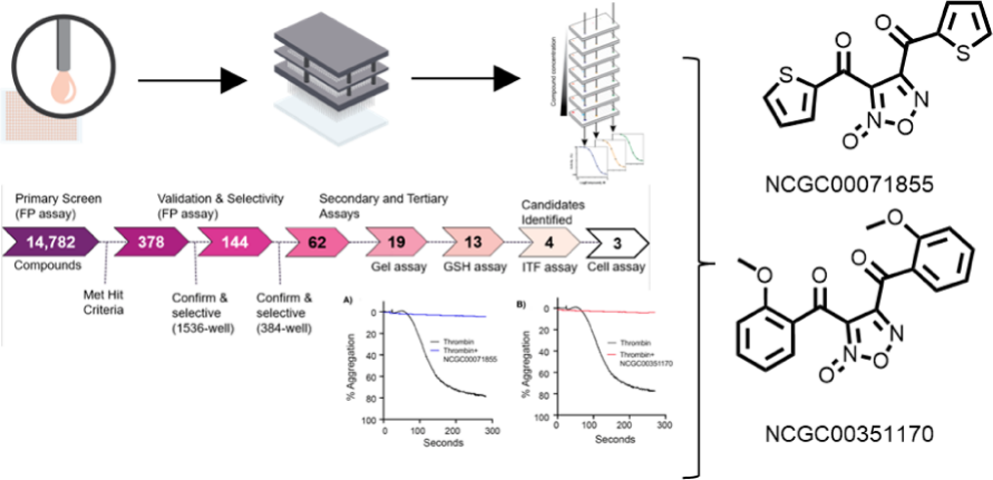

Thrombosis, a key factor in most cardiovascular diseases, is a major contributor to human mortality. Existing antithrombotic agents carry a risk of bleeding. Consequently, there is a keen interest in discovering innovative antithrombotic agents that can prevent thrombosis without negatively impacting hemostasis. Platelets play crucial roles in both hemostasis and thrombosis. We have previously characterized calcium- and integrin-binding protein 1 (CIB1) as a key regulatory molecule that regulates platelet function. CIB1 interacts with several platelet proteins including integrin α_IIb_β_3_, the major glycoprotein receptor for fibrinogen on platelets. Given that CIB1 regulates platelet function through its interaction with α_IIb_β_3_, we developed a fluorescence polarization (FP) assay to screen for potential inhibitors. The assay was miniaturized to 1536-well and screened in quantitative high-throughput screening (qHTS) format against a diverse compound library of 14,782 compounds. After validation and selectivity testing using the FP assay, we identified 19 candidate inhibitors and validated them using an in-gel binding assay that monitors the interaction of CIB1 with α_IIb_ cytoplasmic tail peptide, followed by testing of top hits by intrinsic tryptophan fluorescence (ITF) and microscale thermophoresis (MST) to ascertain their interaction with CIB1. Two of the validated hits shared similar chemical structures, suggesting a common mechanism of action. Docking studies further revealed promising interactions within the hydrophobic binding pocket of the target protein, particularly forming key hydrogen bonds with Ser180. The compounds exhibited a potent antiplatelet activity based on their inhibition of thrombin-induced human platelet aggregation, thus indicating that disruptors of the CIB1- α_IIb_β_3_ interaction could carry a translational potential as antithrombotic agents.

## Introduction

The primary function of platelets is to maintain hemostasis. At the site of vascular injury, platelets adhere to subendothelial proteins such as collagen and the von Willebrand factor. The initial adhesion events rapidly activate platelets, resulting in platelet shape change, granule secretion, and activation of the platelet-specific integrin α_IIb_β_3._^1−3^ Adherent platelets also generate soluble platelet agonists such as thrombin and thromboxane A_2_ (TxA_2_), and the exposure of circulating platelets to these agonists results in their recruitment to the site of injury. Fibrinogen binding to activated integrin α_IIb_β_3_ triggers outside-in signaling, leading to platelet aggregation, stabilization of platelet plug, and clot retraction.^4^ Atherosclerotic plaque rupture also causes platelet plug formation, which underlies several cardiovascular diseases, such as myocardial infarction and stroke. Many antiplatelet drugs such as aspirin, clopidogrel, prasugrel, vorapaxar, and eptifibatide are currently available to prevent thrombotic complications.^5,6^ However, these antiplatelet agents have several limitations including inherent variability in individual responses, developing resistance, and serious bleeding side effects.^7^ Based on the assessment of the current antiplatelet drugs, it appears that the most useful therapeutic approach is to intersect the secondary responses such as integrin outside-in signaling or recruitment of platelets by TxA_2_ and secreted adenosine diphosphate (ADP). Compounds, which can largely preserve the primary hemostatic function of platelets but inhibit secondary response, will be the most desired therapeutic attributes for the treatment of cardiovascular disease.

Calcium- and integrin-binding protein 1 (CIB1) is a ubiquitously expressed, Ca^2+^-binding cytosolic protein, which lacks enzymatic activity and displays a broad functionality in various cellular processes.^8^ CIB1 can regulate the function of various proteins in platelets such as apoptosis signal-regulating kinase (ASK1), p21-activated protein kinase 1 (PAK1), focal adhesion kinase (FAK), and platelet integrin α_IIb_β_3._^9−12^ We previously showed that interaction of CIB1 with α_IIb_ is required for the recruitment of FAK to propagate outside-in signaling.^13^ Subsequently, we also showed that *Cib1*-null platelets show defects in functionality such as aggregation and spreading.^14^*Cib1*-null mice exhibit increased tail bleeding time and delayed arterial occlusion in the FeCl_3_ injury model.^14^ Previous work demonstrated that CIB1 binds to the cytoplasmic domain of the α_IIb_ chain in α_IIb_β_3_ heterodimers following platelet activation.^12,13,15−17^ It has been shown that CIB1 is required for activation of integrin α_IIb_β_3._^18^ Therefore, identification of inhibitors that disrupt the interaction of the α_IIb_ chain with CIB1 could be potential drug candidates to target the platelet function.

Here, we report the development of a high-throughput assay that measures the interaction between α_IIb_ and CIB1 using fluorescence polarization (FP). The FP assay was subsequently utilized in a quantitative high-throughput screening (qHTS) campaign to discover inhibitors of α_IIb_ and CIB1 interaction.^19^ A screen of 14,782 small molecules yielded three structurally distinct inhibitors, two of which were further validated as antiplatelet agents in our platelet aggregation assay. We then employed docking studies complemented by classical quantitative structure–activity relationship (QSAR) analyses to assist in predicting how these ligands might conform to their specific target binding site.^20−22^ These studies are instrumental in predicting the conformations of small-molecule ligands as they interact with specific target binding sites, offering a detailed view of potential drug–target interactions. Our research leverages this technique to shed light on the structural necessities for effective inhibition of key biological targets. Specifically, we concentrated on three distinct compounds: NCGC00071855, NCGC00351170, and NCGC00351154. By examining their interactions within the CIB1-binding pocket, we aim to discern the intricate details of ligand binding, which are pivotal in driving the efficacy of potential inhibitors. This focus on docking studies not only enhances our understanding of molecular interactions but also guides the rational design of new and more effective pharmaceutical agents.

## Results

### Fluorescence Polarization (FP) Assay Development and Optimization

To identify inhibitors of CIB1 and α_IIb_ interaction, we developed an FP assay utilizing a fluorescently labeled (FITC) peptide from the cytoplasmic domain of α_IIb_ (F-α_IIb_) that interacts with CIB1. When excited by polarized light, free F-α_IIb_ quickly tumbles in the solution, causing the emitted light to exhibit lower polarization, *versus* when F-α_IIb_ is in complex with CIB1, leading to an increase in polarization from the emitted light. Therefore, if a small molecule can disrupt the interaction, we will observe a decrease in the measured FP signal.

**Figure 1:**
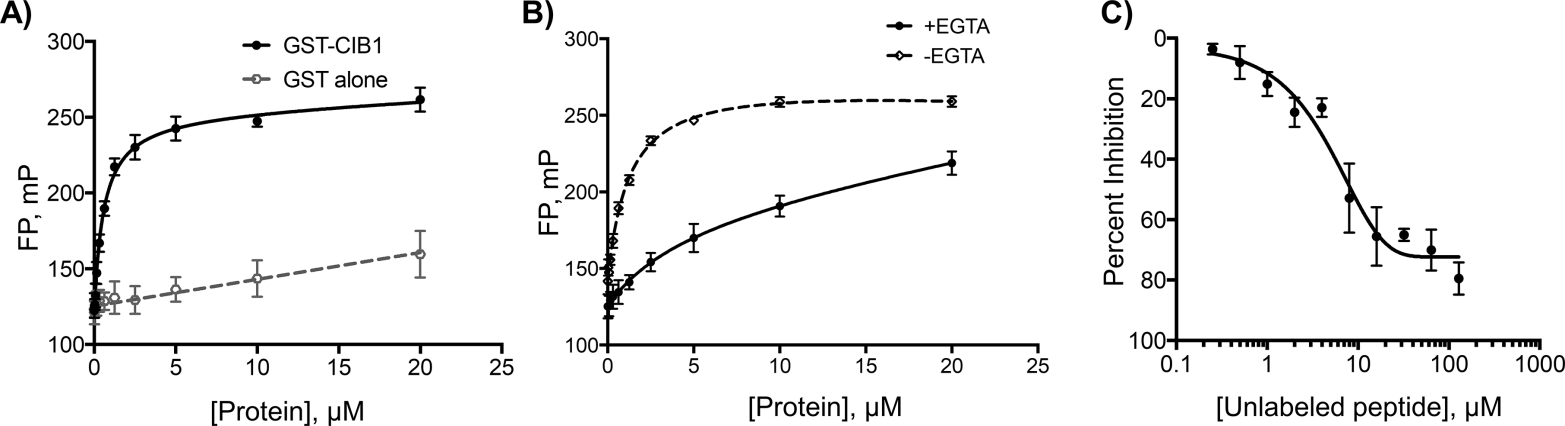
Development and characterization of the FP assay to identify inhibitors of CIB1. (A) Various concentrations of GST-CIB1 or GST alone titrated with 100 nM F-α_IIb_ peptide. The binding of protein and peptide was measured by an increase in milli polarization. (B) Various concentrations of GST-CIB1 titrated with 100 nM of F-α_IIb_ peptide in the presence and absence of 5 mM EGTA. (C) Inhibition of GST-CIB1 (1 μM) and F-α_IIb_ peptide (100 nM) by unlabeled αIIb peptide pre-incubated for 1 h. The percentage of inhibition was calculated using F-αIIb peptide milli polarization as 100% inhibition and F-α_IIb_ peptide + GST-CIB1 milli polarization as 0% inhibition. Data represent ± SEM of three independent experiments.

First, we incubated 100 nM F-α_IIb_ with varying amounts of GST-tagged recombinant CIB1 (GST-CIB1) in a 96-well-plate, where we observed binding in a concentration-dependent manner (Figure 1A). To show that this interaction is specific, we incubated F-α_IIb_ with GST alone, where binding was not observed (Figure 1A). Previous studies have shown that the cytoplasmic domain of α_IIb_ binds to CIB1 in a calcium-dependent manner.^16^ Therefore, to further confirm the specificity of binding of F-α_IIb_ to GST-CIB1 in the FP assay, we used egtazic acid (EGTA) to chelate Ca^2+^. As shown in Figure 1B, the F-α_IIb_ peptide binds weakly to GST-CIB1 when in the presence of EGTA, suggesting that the CIB1:F-α_IIb_ interaction is Ca^2+^-dependent. We then tested the ability to disrupt the interaction by performing a competitive binding assay using increasing doses of unlabeled α_IIb_ peptide to a fixed concentrations of GST-CIB1 (1 μM) and the F-α_IIb_ peptide (100 nM). Unlabeled α_IIb_ peptide successfully blocked the binding of F-α_IIb_ to GST-CIB1 with a half maximal inhibitory concentration (IC_50_) of 11.2 μM (Figure 1C).

We then moved to miniaturize the assay by testing various assay conditions in 384-well format, such as varying concentrations of probe F-α_IIb_ and GST-CIB1, incubation times and compatibility with DMSO. All experiments were carried out in 50 μL volume (see the Materials and Methods Section for details). The titration of the probe indicated a strong signal to background (S/B) ranging from 47 to 11 at the probe concentrations from 125 to 31 nM, respectively (Figure S1A). The relative binding affinity between F-α_IIb_ and CIB1 was determined by incubating the probe (50 nM) with varying concentration of GST-CIB1 for 15, 30, 60, and 90 min (Figure S1B). The extended incubation times had little effect on the protein:peptide interaction. Using the 30 min time-point, the half maximal effective concentration (EC_50_) of CIB1 was ∼0.2 μM (Hill Slope = 1.04), leading to an EC_80_ of 1.5 μM. EC_80_ or EC_90_ is used as a guideline concentration to set up a screening assay with well-balanced strong signal and the ability for a small molecule to disrupt the interaction.^23^ Because the screening libraries are dissolved in DMSO, we tested the compatibility of the assay at various percentages of DMSO (from 10 to 0.625%), determining that an assay tolerance was up to 5% (v/v) DMSO when incubated with 1 μM protein and 50 nM probe (Figure S1C).

Next, we tested whether the unlabeled peptide could inhibit the interaction between F-α_IIb_ and CIB1 under the selected conditions in the 384-well format. We performed a titration of unlabeled-α_IIb_ (2 mM, 1:2 dilution, 16-point, *n* = 3) with F-α_IIb_ (final concentration of 50 nM) and GST-CIB1 (final concentrations of 1 or 2 μM), where we observed an IC_50_ value of ∼2.7 μM (Figure S1D). Of note, at unlabeled peptide concentrations >25 μM, we noticed a “hook effect” and excluded those points, possibly due to aggregation or quenching of signal.

To discern nonspecific inhibitors and FP-interfering molecules, we designed a scrambled-peptide counter screen. The ability of the labeled scrambled-peptide (F-scrambled) to bind to GST-CIB1 was examined using conditions similar to the above binding affinity experiments. An EC_50_ value of 1.6 μM was observed (Figure S1E), indicating the binding of the F-scrambled to CIB1, although the affinity is ∼8-fold weaker compared to the native peptide (F-α_IIb_; 1.6 *vs* 0.2 μM).

We further miniaturized the assay to 1536-well format (Table S1) by testing F-α_IIb_ concentrations at 25, 50, and 100 nM in a four μL assay volume, where we observed S:B of 6.8, 9.5, and 18, respectively, versus buffer only solution (Figure S2A). We chose 100 nM F-α_IIb_ due to the strong S/B, along with 1 μM CIB1-GST based on the 384-well assay statistics. We then tested the activity of the unlabeled α_IIb_ peptide, resulting in an IC_50_ value of ∼12 μM, ∼4-fold higher than the ∼3 μM IC_50_ observed in the 384-well format (Figure S2B). Similar to the 384-well format, we observed a “hook effect” at concentrations >35 μM. The assay performance was acceptable, with a Z′-factor of 0.56 and a signal window (ΔmP) of 63, proving adequate for conducting an HTS.

### Quantitative High-Throughput Screening and Compound Triage

After finalizing our HTS assay conditions, we performed a pilot screen against the LOPAC^1280^ (Library of Pharmacologically Active Compounds) compound library, arrayed as a dilution series of 5-plates in qHTS format (10 mM, 1:5 dilution), resulting in a final assay concentration of 457 nM to 114 μM. The pilot screen performed well, yielding a mean Z′-factor of ∼0.8 and an increased signal window of ∼96 ΔmP. The unlabeled-α_IIb_ peptide was used as an intraplate control with an IC_50_ of 8.6 μM and a minimum significant ratio (MSR) of 2.7 (Figure S2C), indicating good reproducibility.^19,24^ From the pilot screen results, we decided to use 4,5,6,7-tetrabromobenzotriazole (TBB, NCGC00092352; a double-digit micromolar inhibitor) as our intraplate control instead of the unlabeled-α_IIb_ peptide for subsequent screening (Figure S2D).

**Figure 2:**
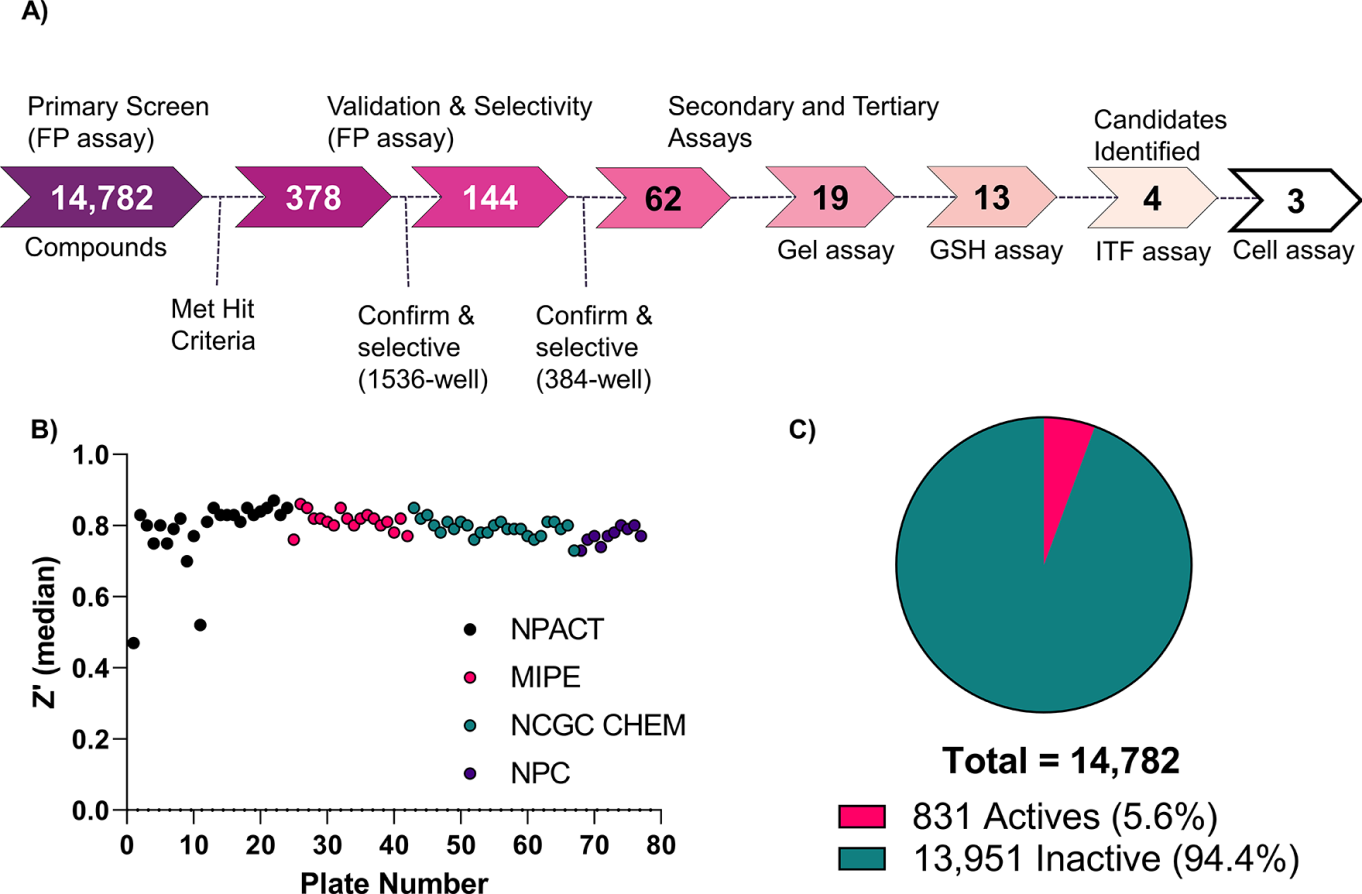
Screening and validation of potential hits. (A) Schematic representation of the HTS assay triage summary. (B) CIB1 qHTS plate performance as *Z*′ values for each library screened. (C) CIB1 qHTS hit rate.

Based on the positive results of our LOPAC^1280^ screen, we then tested our in-house collection of approved, investigational, and annotated compound libraries (see Materials and Methods Section). As shown in Figure 2A, a total of 14,782 compounds were screened in dose–response, with a cumulative *Z*′ of 0.79 ± 0.06 (Figure 2B) and an intraplate control TBB IC_50_ of ∼10.7 ± 3.5 μM (MSR = 2.5). All substances tested in the qHTS format yielded concentration–response curves (CRC) from the primary screen, enabling us to prioritize active samples (Figure S3). Because of the low hit rate (typical of PPI),^25^ we had an initial low bar for selection, with any compound exhibiting a negative curve class (Figure S3). Using these criteria, 831 compounds (5.6% hit rate) were identified as potential inhibitors (Figure 2C and Table S2). We then applied structural filters to eliminate electrophiles and other frequent hitters,^20,26−31^ resulting in 415 compounds, of which 378 were sourced for testing against the primary and counter screens (Figure 2A and Tables S2 and S3).

### Hit Characterization

Of the 378 compounds, 275 were confirmed (73%) using the primary screening assay, but 131 exhibited activity in the counter screen, leaving 144 with selectivity toward α_IIb_ (Table S3), with IC_50_ values ranging from 0.4 to 100 μM. We selected 108 of the 144 compounds for further testing using the more sensitive 384-well-plate format against both the F-α_IIb_ and F-scrambled peptides. Of the 108, 93 exhibited inhibition versus the α_IIb_ peptide (86%), but 25 exhibited activity toward the scrambled peptide (plus an additional 6 that were inconclusive), leaving 62 selective toward α_IIb_ (Table S4; defined as exhibiting a negative curve class and/or IC_50_ ratio of >3-fold and/or >2-fold efficacy) with IC_50_ values ranging from 0.3 to 120 μM.

**Figure 3:**
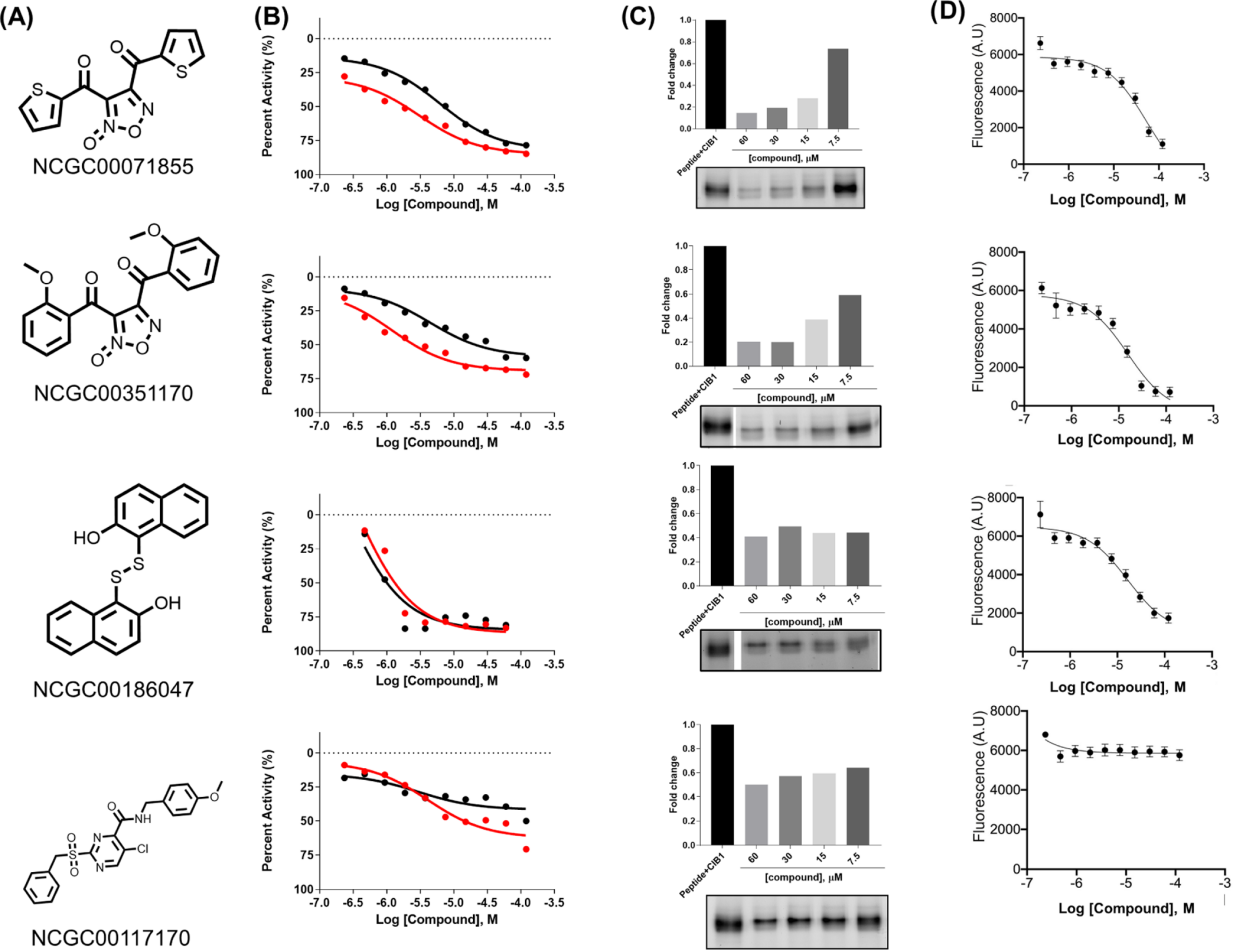
Characterization of candidate inhibitors. (A) Structures of candidate inhibitors from screening campaign. (B) Dose–response curves of four selected compounds (from 0.23 to 120 μM) with 100 nM F-α_IIb_ peptide and 1 μM GST-CIB1 with (red circle) and without (black circle) GSH. (C) In-gel assay results for the selected compounds. Compounds tested at indicated concentrations were pre-incubated with 1 μM GST-CIB1 for 1 h. After incubation, 400 nM F-α_IIb_ peptide was added to each reaction and incubated for 15 min. Samples were resolved using SDS-PAGE gels and bound F-α_IIb_ peptide visualized by fluorescent Bio-Rad gel imager. (D) Tertiary validation and characterization of selected compounds by ITF assay. Dose–response of four selected compounds with 10 μM F-α_IIb_ peptide and 10 μM GST-CIB1.

To determine the validity of the hits, we developed a low-throughput gel-based binding experiment as a secondary assay to assess the compound’s activity and remove FP assay artifacts. The gel-based assay offers the advantage of being able to visualize the disruption of the CIB1 interaction by monitoring the labeled peptide, allowing for dose-dependent quantification of the inhibition. Because of the labor-intensive nature of the protocol, we selected 19 compounds for further characterization (Table S4). In this assay, 1 μM GST-CIB1 was incubated with 400 nM F-α_IIb_ peptide in the presence or absence of test compound. An example gel of weakly active (low inhibition) and active (exhibiting inhibition) compounds is shown in Figure S4A,B. Out of the 19 compounds tested, 14 (or 74%; Figures 3C and S5A–N) inhibited the GST-CIB1:F-α_IIb_ interaction in the gel assay.

Inspecting the 14 candidate inhibitors, we flagged two disulfide containing compounds, NCGC00091563 (thiram) and NCGC00016000 (disulfiram), as potential false positives due to their ability to cross-link with cysteines.^32^ To determine if our hits were covalently interacting with active site cysteine, we tested 13 of the 14 compounds in the presence of the physiological reducing agent glutathione (GSH) in the FP assay (Table S5).^32^ First, we confirmed that the addition of GSH (ranging from 10 to 100 μM) had no effect on the interaction between F-α_IIb_ and GST-CIB1, as shown in Figure S6A. Next, we examined both disulfide containing compounds, where we observed IC_50_ shifts of 8.3- and 8.5-fold for thiram and disulfiram, respectively, indicating that they were affected by the presence of GSH and interacting with the cysteines of CIB1 (Figures 3B and S6B,C). Of the remaining 11 candidate inhibitors, four, NCGC00262908 (ticlatone), NCGC00178879 (thimerosal), NCGC00181776 (dipentamethylenethiuram disulfide), and NCGC00189495, produced a right-shift in potency similar to thiram and disulfiram. All these compounds contained a free thiol group except for NCGC00186047 (IPA-3), which contains a disulfide moiety. IPA-3 was one of the seven compounds where no shift was observed (Figure S6D–N).

To further characterize the hit compounds, we tested four of the above seven in an orthogonal intrinsic tryptophan fluorescence (ITF) assay^33,34^ with unlabeled α_IIb_ peptide and CIB1 lacking the GST tag. The α_IIb_ peptide contains the amino acid tryptophan in its sequence, enabling us to detect its signal when excited at 295 nm and when emission was measured at 355 nm. Due to the environmental sensitivity of tryptophan, the interaction of the peptide with CIB1 induces a change in the intrinsic fluorescence signal. As shown in Figure 3D, three of the four compounds tested (IPA-3, NCGC00351170, and NCGC00071855) produced a concentration-dependent intensity change in the ITF assay in the presence of CIB1 and α_IIb_, with IC_50_ values of 16.6, 15.6, and 53.1 μM respectively, following a similar ranking observed in the FP assay (Table S5).

Considering their structural similarity stemming from their shared furoxan chemotype, we opted to further explore NCGC00071855 and NCGC00351170. We conducted a search within our in-house compound library and identified 10 analogs, testing them in the 384-well FP assay. The active analogs revealed a range of potencies with IC_50_ values from 4.2 to 20.7 μM. Additionally, a structural activity relationship (SAR) was observed within this set (see Table S6 and Figure S7A–J).

### *In Vitro* and *In Silico* Target Engagement Studies

Determining a direct measurement of a molecule’s binding interaction with its target is a vital parameter for characterizing a chemical probe. We employed microscale thermophoresis (MST) for this purpose, leveraging its sensitivity and capacity to deliver quantitative data on the binding interaction.^35^ For the MST experiments, we chose to pursue NCGC00351170 and an inactive analog NCGC00351154. We began by testing increasing amounts of the compound(s) and incubating them with the fluorescently labeled CIB1-(His)_6_, followed by the detection of the MST traces on the NT automated instrument (for more details see Materials and Methods Section). Both compounds exhibited binding to CIB1, with equilibrium dissociation constants (*K*_d_) of 2.9 and 6.7 μM, respectively (Figure S8A). While we were encouraged by the *K*_d_ value of NCGC00351170, we were surprised by NCGC00351154 exhibiting only a 2.3-fold difference in binding affinity, as it was inactive in the qHTS assay (over the concentration range tested). We were able to rule out compound interference with the histidine tag on CIB1 and/or the fluorophore used for the MST assay by testing the compounds with a fluorescently labeled hexa-histidine peptide (Figure S8B).

**Figure 4:**
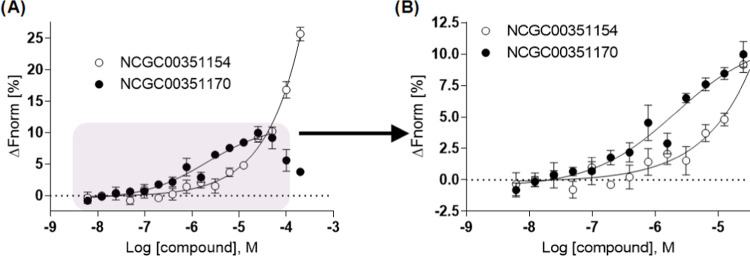
MST competitive binding assay. We selected 200 nM F-α_IIb_ and a CIB1 concentration of 4 μM and tested both compounds. (A) For NCGC00351170 (●), we observed an EC_50_ value of 2.1 μM, whereas for NGC00351154 (○), in the presence of peptide, we observed an EC_50_ of 29.0 μM, a 13.8-fold difference between the two compounds (*n* = 3/concentration). Panel (B) highlights the concentration range in which we observe different activities between the two compounds.

We sought to resolve this discrepancy by performing a competition binding study similar to the conditions of the FP HTS assay by utilizing the F-α_IIb_ probe. First, we measured the binding of F-α_IIb_ to the same CIB1-(His)_6_ protein as before and calculated a *K*_d_ of 2.6 μM (Figure S8C). We selected 200 nM F-α_IIb_ and a CIB1 concentration of 4 μM (1.5 × *K*_d_)^36,37^ and tested both compounds. For NCGC00351170, we observed a similar EC_50_ value of 2.1 μM (*vs* ∼4 μM in the 384-well FP assays), whereas for NGC00351154, in the presence of peptide, we observed a right-shift of 4.3-fold, from 6.7 to 29.0 μM (Figure 4A,B). This 13.8-fold difference between the two compounds appears to be in better agreement with what we observed in the HTS assay, providing further confidence that NCGC00351170 is disrupting the interaction.

**Figure 5:**
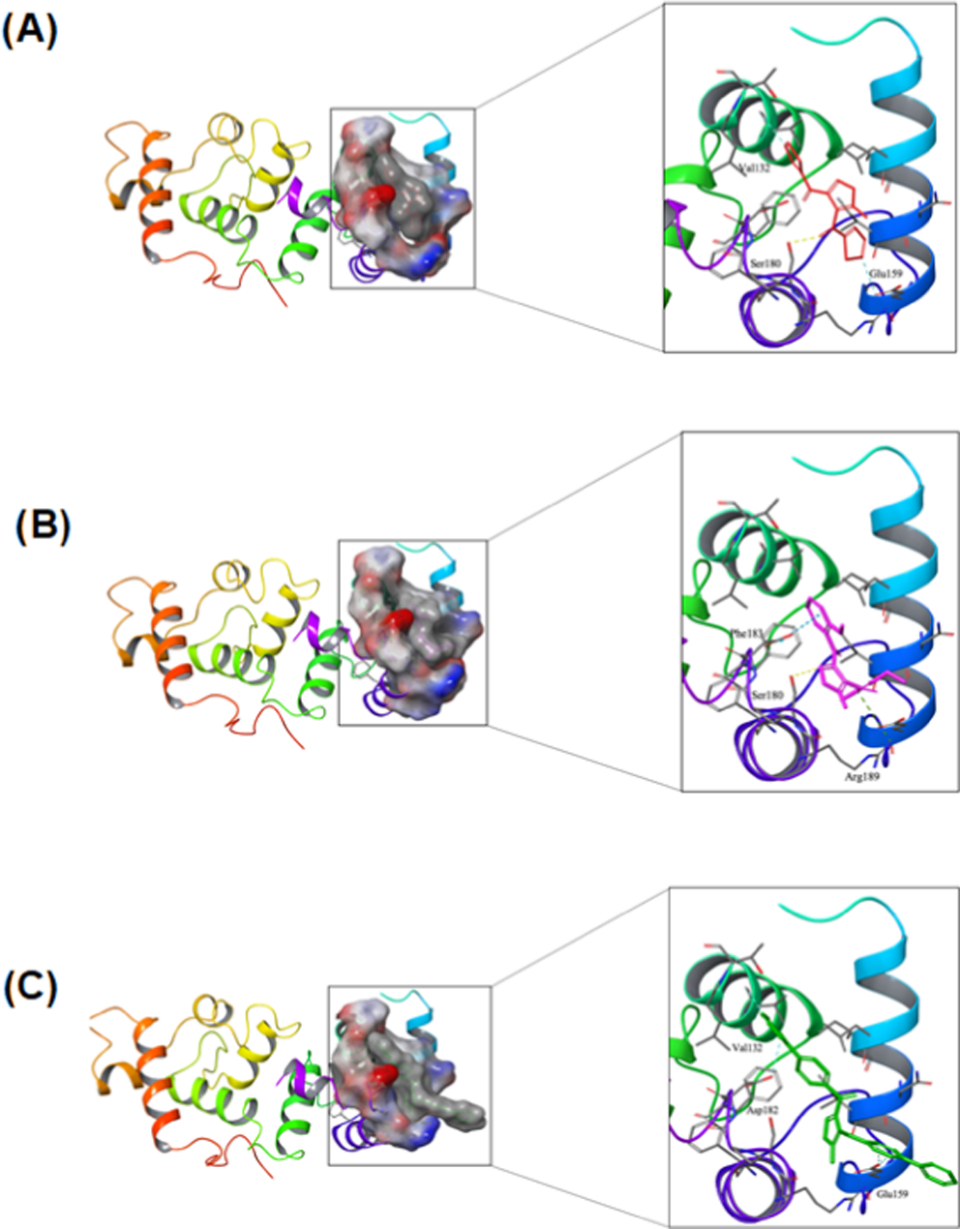
Surface model showing docking pose of (A) NCGC00071855–04 (red) and (B) NCGC00351170 (pink). (C) Surface model showing the docking pose of NCGC00351154 (green).

In addition to classical QSAR studies, docking studies have been extensively employed in modern pharmaceutical sciences due to their ability to predict the conformation of small-molecule ligand interaction with the target binding site. To decipher the structural prerequisites for inhibition, we subjected NCGC00071855, NCGC00351170, and NCGC00351154 to docking within the CIB1-binding pocket. As depicted in Figure 5A,B, both NCGC00071855 and NCGC00351170 fit well in the hydrophobic binding pocket, establishing H-bond interactions with Ser180. Furthermore, NCGC00071855 exhibits an aromatic H-bond interaction with Val132 and Glu159, while NCGC00351170 engages in Pi–Pi interactions with Phe183 and Pi–cation interactions with Arg189. Conversely, NCGC00351154 demonstrates aromatic H-bond interactions with Val132, Glu159, and Asp182. However, its relatively substantial size and considerable solvent exposure contribute to its low ligand efficiency. Additionally, NCGC00351154 is rigid, and binding pocket complementarity, which could account for its diminished activity (Figure 5C). This interpretation is reinforced by binding affinity score predictions obtained from LigandScout 4.4,^38^ yielding a score of −15.62 for NCGC00071855 and a slightly improved score of −16.11 for NCGC00351170. These scores align with the observed outcomes in the biological experiments. NCGC00351154 received a binding affinity score prediction of −12.16. These findings strengthen the hypothesis that hydrogen bonding with Ser180 could potentially be a driving factor behind the increased activity. Upon additional experimental validation, this insight may guide the rational optimization of compounds toward enhanced potency. These findings could provide a basis for a future campaign on the structure–activity relationship (SAR) of this chemotype.

### ADME Properties

To demonstrate their potential for further development, we assessed the *in vitro* ADME properties of the hit compounds NCGC00071855 and NCGC00351170, along with selected analogs, as detailed in Table S7. These compounds exhibited moderate solubility and PAMPA permeability, but their poor RLM stability necessitates further optimization before they can be evaluated in *in vivo* models.^39−43^

### *Ex Vivo* Characterization of Hit Compounds in a Human Platelet Aggregation Assay

A classical challenge in target-based drug discovery is moving from biochemical to cellular assay and the essential step of determining if the compounds of interest can perform in a cellular context. After characterization of the compounds in a series of biochemical assay formats, our next step was to assess their activity in a cellular environment. Our previous studies have shown that the interaction of the cytoplasmic domain of integrin α_IIb_β_3_, a key regulator of platelet aggregation, with CIB1, is involved in downstream signaling in platelet activation and aggregation. We sought to determine whether our candidate compounds were inhibiting this interaction by evaluating them for platelet function in a human platelet aggregation assay. We prepared platelet-rich plasma (PRP) and washed the platelets as previously described^44^ and then performed a platelet aggregation assay using the washed platelet suspensions (2 × 10^8^/platelets/mL) followed by the recording of aggregation traces (see Materials and Methods Section). To induce platelet aggregation, 0.03 U/mL of thrombin, a physiological agonist of platelet activation, was used.

**Figure 6:**
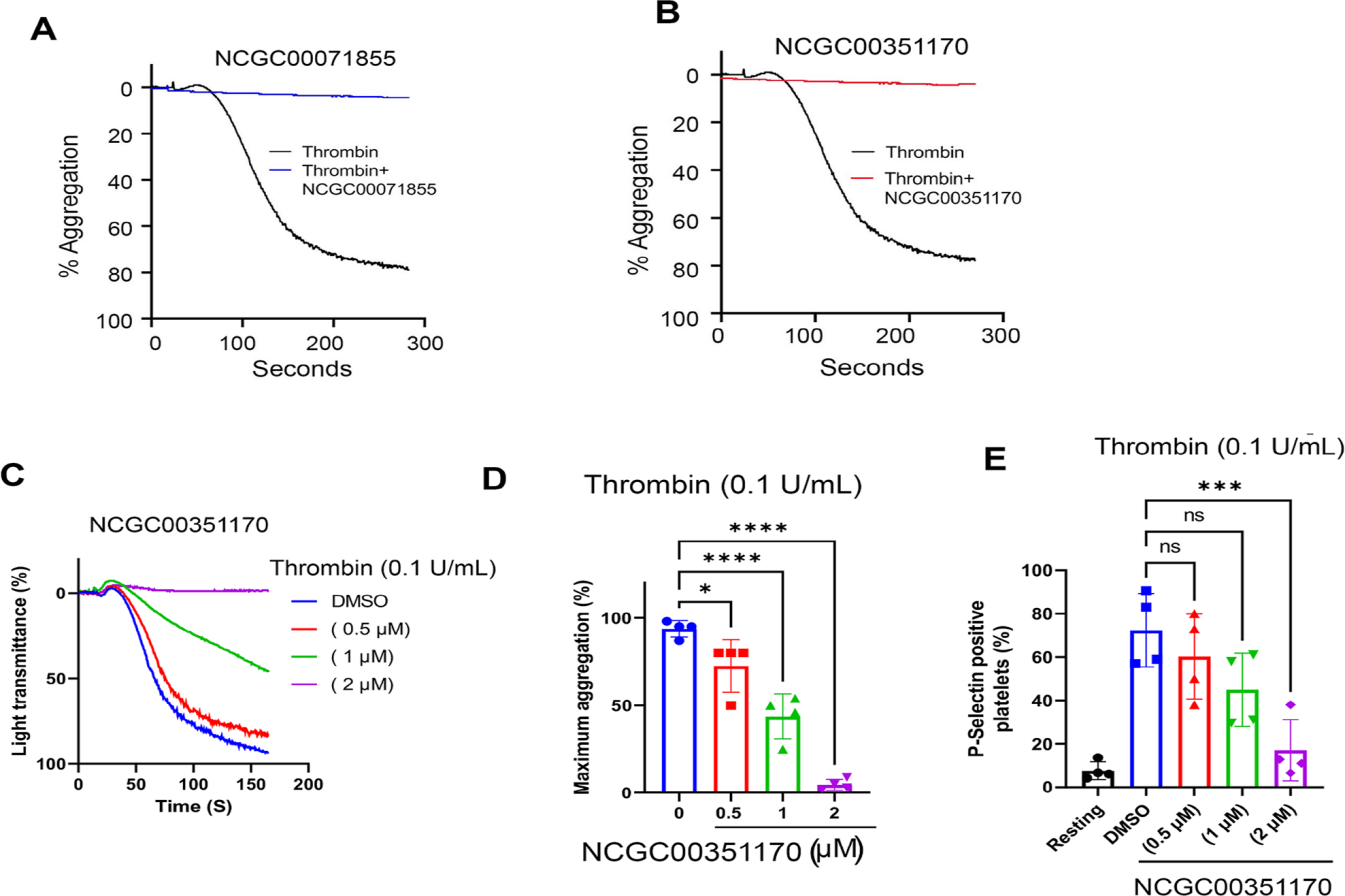
Effect of compounds on human platelet activation. Representative platelet aggregation tracings of washed human platelet suspensions pretreated for 30 min at 37 °C with vehicle (DMSO) or compounds at indicated concentrations followed by activation with thrombin (0.03–0.1 U/mL). Panel (A): NCGC00071855. Panel (B–D): NCGC00351170. Panel (E): CD62P exposure was in the presence of NCGC00351170. Data represents mean ± SEM, *n* = 4 individual experiments; ns = non significant *P < 0.05, ***P < 0.001, ****P < 0.0001.

As shown in Figure 6A,B, both compounds were initially tested at a single concentration of 10 μM and were successful in 97–99% inhibition of platelet aggregation. We then tested the analogs of both hits in the same assay, where 7/10 exhibited strong inhibition (>75%), while the three compounds that exhibited poor to no inhibition correlating with the weakest potencies in the FP assay (Tables 1, S6 and Figure S9A–E). These findings indicate that through the utilization of this FP assay, we successfully identified inhibitors of platelet function, which may warrant further assessment for their potential as antiplatelet agents. We next tested various doses of NCGC00351170 and found that it dose-dependently inhibited platelet aggregation with 2 μM being the lowest concentration required to achieve >95% inhibition (Figure 6C,D). Finally, we evaluated the effect of NCGC00351170 on P-selectin (CD62P) exposure (a marker for platelet activation) and observed a dose-dependent inhibition of platelet activation, confirming the effect of NCGC00351170 on platelet function (Figure 6E).

**Table 1:** Compounds were Tested at a Single Concentration of 10 μM (*n* = 3) and Percent Inhibition for Platelet Aggregation was Determined^a^

		FP assay	aggregation assay
sample ID	PubChem CID	IC_50_ [μM]	inhibition (%)
NCGC00351170–01	86242782	4.19	94
NCGC00351166–01	20328617	4.26	77
NCGC00064079–03	286532	4.53	99
NCGC00351163–01	1388811	6.68	96
NCGC00351167–01		6.68	84
NCGC00351164–01		7.26	98
NCGC00351162–02	289811	7.50	97
NCGC00351168–02	227326	7.85	97
NCGC00351160–01		20.7	49
NCGC00351154–01	286556	NC	16
NCGC00354607–01		NC	14

a Inhibitory activity correlated well with the potency observed in the FP assay.

## Discussion

Disrupting protein–protein interactions (PPIs) is one of the most difficult strategies for drug development. Having identified the CIB1 protein and accompanying α_IIb_ peptide as a PPI with potential therapeutic consequences, we miniaturized and optimized an FP assay to identify inhibitors of this interaction. Recognizing the cost-effectiveness, speed, and scalability advantages of the FP format, we chose it for this PPI interrogation study, despite it being less commonly used than some immuno- or bead-based technologies. We were able to rapidly screen a diverse set of 14,782 compounds, taking candidate hits through a series of secondary, tertiary, and orthogonal assays, to arrive at three candidate inhibitors: IPA-3, NCGC00071855, and NCGC00351170. While there is evidence that IPA-3 (a well-studied compound from the LOPAC^1280^ compound library) could interact with the CIB1-α_IIb_ complex, its identification as an unsuitable probe led us to pause any further characterization,^9,45−47^ enabling us to focus our efforts on NCGC00071855 and NCGC00351170.

NCGC00071855 (CID 573747) is a well-characterized molecule, with over 925 biological test results documented in PubChem. It has been previously published for targets, including antischistosomiasis/thioredoxin glutathione reductase (TGR),^48^ sperm motility,^49^ and glutathione peroxidase 4 (GPX4).^50^ Although the CIB1-binding pocket contains residues similar to those present in the active site of these targets, the CIB1 pocket lacks cysteine residue and enzymatic activity, suggesting potential similarities in the interactions with NCGC00071855 but distinct mechanisms of action or biological effects. Our screening collection includes previously synthesized National Center for Advancing Translational Science (NCATS) compounds, leading to the identification of our third candidate inhibitor, NCGC00351170.^51^ This compound, a structural analogue of NCGC00071855, has demonstrated a potency profile comparable to its predecessor in our assays. It is worth noting that NCGC00351170, like other furoxans and their analogs, is known to undergo metabolism, producing nitric oxide and nitrile oxide electrophiles and inducing ferroptosis.^52^ These reactive species can covalently bind to and inactivate selenocysteine-containing proteins. We do not envision such a prodrug type mechanism being involved in their interference in the binding of CIB1 to α_IIb_. However, achieving selectivity for disrupting the interaction between CIB1 with α_IIb_ will necessitate extensive further optimization. The recognition of comparable potency profiles and the understanding of the metabolic pathways associated with this chemotype can guide future optimization efforts to develop more selective inhibitors for CIB1 modulation. Considering the cytotoxic role of diacylfuroxans, it will be important to carefully assess the cytotoxic effect of these hits and modify the structures to minimize such effects without compromising efficacy.

Our docking studies revealed that NCGC00071855 and NCGC00351170 firmly occupy the hydrophobic binding pocket, establishing hydrogen bonds with Ser180. Furthermore, NCGC00071855 formed aromatic hydrogen bonds with Val132 and Glu159, while NCGC00351170 engaged in Pi–Pi interactions with Phe183 and Pi–cation interactions with Arg189. In contrast, NCGC00351154 faced challenges due to its larger size and significant solvent exposure, resulting in reduced ligand efficiency. Its rigidity and less favorable binding pocket complementarity likely contributed to its decreased activity. Binding affinity score predictions supported these findings, underlining the potential role of hydrogen bonding with Ser180 in enhancing activity. However, we did not see any cysteine residue potentially interacting with the compounds, suggesting that these compounds may not covalently modify CIB1 as they are known to modify selenocysteine and thus may exert a reversible inhibition. After further experimental validation of their reversibility and analysis of half-life can offer valuable insights for the purpose of strategically enhancing the compound’s potency.

CIB1 has been shown to interact with almost two dozen of proteins, which play significant roles in cell adhesion and migration, cell cycle, and cytoskeletal rearrangements.^8,10,53^ Our compounds presented here are the product of a specific screen developed to inhibit the interaction of CIB1 with α_IIb_. However, it cannot be ruled out that these compounds will not interfere with the ability of CIB1 to interact with its other binding partners. Of note, it has been shown that CIB1 interacts with several protein kinases and inhibits their activity.^44,53^ In addition to cardiovascular diseases, CIB1 is considered a target for a number of other diseases such as cancer and neurodegenerative diseases.^54−56^ In an attempt to identify CIB1 inhibitors to inhibit cancer cell survival, Puhl et al. used a random peptide display library screening and identified a linear peptide that specifically binds CIB1 and inhibits its function in cancer cells.^57^ To overcome issues with stability and potency, a cyclic peptide was identified and characterized for its effect on triple-negative breast cancer cell proliferation and survival.^58^ Considering the chemical nature of the small molecules reported here, we believe that this study will act as a starting point for further characterization and validation of their functions in downstream assays including animal models and offer a separate avenue for identifying inhibitors of CIB1 that could be used as pharmacological interventions for various human diseases.

## Materials and Methods

### Reagents

Glutathione sepharose beads were obtained from GE Healthcare. Black 384-well plates were purchased from Thermo Fisher Scientific. Unlabeled and fluorescein isothiocyanate-conjugated α_IIb_ peptides, as well as scrambled control peptides, were custom synthesized from Peptide 2.0, at >95% purity. All peptides were dissolved in DMSO, aliquoted, and stored at −80 °C.

Sequence of unlabeled α_IIb_ peptide: acetyl-LVLAMWKVGFFKRNRK (purity is >95.00%); sequence of F-α_IIb_ peptide: acetyl-LVLAMWKVGFFKRNRK-FITC (purity is 95.83%); and sequence of F-scrambled α_IIb_-peptide: acetyl-RKLFVKVMFWRLNGAK-FITC (purity is 95.37%).

### Expression and Purification of CIB1

The cloning and expression of CIB1 as glutathione S-transferase fusion protein (GST-CIB1) has previously been reported.^12,59^ A single colony of BL-21 plus RIL cells transformed with GST-CIB1 expression construct or empty vector (pGEX-4T) was grown into log phase in the presence of 100 μg/mL of ampicillin, and protein expression was induced by incubation with 100 μM isopropyl-β-d-thiogalactoside (IPTG) for 3 h at 37 °C. After incubation cells were harvested, resuspended in 20 mM Tris pH 8.0, 500 mM NaCl, 1 mM phenylmethylsulfonyl fluoride, 10 μg/mL of aprotinin, 10 μg/mL of leupeptin, and 5 mM DTT and lysed using French Press. The CIB1-GST fusion protein or free GST was purified using glutathione-affinity chromatography (GE HealthCare, Inc.) followed by elution with 10 mM reduced glutathione. CIB1-GST fusion protein as well as GST were extensively dialyzed for 18 h in 5 mM HEPES pH 7.5, 125 mM NaCl, 5 mM CaCl_2_, and 0.25 mM DTT. The proteins were concentrated to 10 mg/mL, aliquoted, flash-frozen and stored at −80 °C until further use. The purity of the protein preparation was analyzed by SDS-PAGE and Coomassie staining (Figure S10). The molecular weight of GST-CIB1 was 48 kDa, whereas the molecular weight of GST alone was 26 kDa. CIB1-(His)_6_ protein, used in some experiments, was purified using a Ni-NTA resin column following manufacturer instructions (Fisher Scientific).

### Compound Screening

The LOPAC^1280^ collection was purchased from Sigma-Aldrich. Four NCATS internal libraries screened in this study are the NPACT (5,099 compounds), MIPE v4 (1912 compounds),^60^ the NCATS Pharmaceutical collection (2,816 compounds),^61^ and the NCATS Chemistry collection (7448 compounds). The NCATS Chemistry collection is a library consisting of internally synthesized compounds. For a full list of compound structures, see PubChem Assay Identifier (AID) 1508620.

All compounds were initially sourced from the National Center for Advancing Translational Sciences (NCATS)/National Institutes of Health (NIH). All compounds were subjected to quality control by LC/UV, liquid chromatography/mass spectrometry (LC/MS), or High-resolution MS, with all compounds exhibiting >95% purity by peak area or *m*/*z*.

### Fluorescence Polarization (FP) Assay in 96- and 384-Well Format

Initial experiments for FP assay development were set up in a 96-well-plate with a total volume of 200 μL. FITC-labeled α_IIb_ peptide (F-α_IIb_) and GST-CIB1 were incubated at indicated concentrations for 15 min at room temperature (RT), and the FP signal was measured with PerkinElmer Victor 3 plate reader.

FP assay optimizations were carried out in 384-well solid-bottomed black plates (Greiner Bio-One, Monroe, NC). To begin, we prepared F-α_IIb_ in assay buffer (5 mM HEPES pH 7.4, 125 mM NaCl, 5 mM CaCl_2_, 0.01% Tween 20, and 0.25 mM DTT prepared fresh) at a starting concentration of 1 μM and performed a 1:2 dilution (20 μL assay volume), in addition to a buffer only control sample. Samples were read for FP (*E*_x_ = 480(20)/*E*_m_ = 540(25) S and P; FITC Dichroic mirror) on a ViewLux CCD imager and used the S-channel RFU’s to determine S:B, choosing 50 nM (S:B ∼20; Figure S1A). For determination of α_IIb_ binding to GST-CIB1, 10 μL F-α_IIb_ (100 nM, final concentration) was incubated with various concentrations of GST-CIB1 protein (final concentration of 0–20 μM) in 10 μL assay buffer at RT in dark for indicated time points before measuring FP.

To test the activity of the unlabeled peptide, 15 μL of CIB1-GST (final concentrations of 1 μM) in assay buffer was dispensed into a 384-well plate, followed by the transfer of 1 μL of unlabeled α_IIb_ peptide (2 mM, 1:2 dilution, 16-point, *n* = 3, in DMSO; final assay concentration range of 3.05 nM to 100 μM). Samples were incubated at RT for 15 min followed by a 5 μL addition of F-α_IIb_ (final concentration of 50 nM). Plates were centrifuged at 1000 rpm (164*g*) for 15 s, incubated at RT for 1 h, and read for FP using the above detection settings. Data were normalized against protein-probe mixture, no-protein controls, and the resulting percent inhibition data were fitted to a 4-parameter Hill equation using GraphPad Prism software (version 9.1).

### FP Assay in 1536-Well Format

Protein (3 μL of CIB1-GST, final assay concentration 1 μM) or assay buffer (5 mM HEPES pH 7.4, 125 mM NaCl, 5 mM CaCl_2_, 0.01% Tween 20) was dispensed into a 1,536-well solid-bottom black plate (Greiner Bio-One, Monroe, NC). Forty-six nL of compound (final assay concentration of 457 nM to 114 μM) was transferred *via* Wako Pin-tool (Wako Automation, Richmond, VA). Samples were incubated at RT for 15 min followed by a 1 μL addition of F-α_IIb_ (final concentration of 100 nM). Samples were centrifuged for 15 s at 1000 rpm (164*g*), followed by a RT incubation for 15 min, then read for FP (Table S1).

### qHTS Data Analysis and Statistics

Data from each assay were normalized plate-wise to corresponding intraplate controls (DMSO neutral control and assay buffer positive control as noted). The same controls were used for the calculation of the *Z*′ factor, a measure of assay quality control.^62^ Concentration–response curves (CRCs) were fitted and classified as previously described, categorized into four classes as shown in Figure S3: complete response curves (class 1), partial curves (class 2), single point actives (class 3), and inactives (class 4).^19,63,64^ All CRCs were fitted as previously described, and IC_50_ values were calculated using in-house software or GraphPad Prism (sigmoidal dose–response variable slope). Minimum significant ratio (MSR),^24^ a statistical parameter that characterizes the reproducibility of potency estimates from *in vitro* concentration–response (CRC) assays, was used to assess the performance of our intraplate controls. The chemical structures were standardized using the LyChI (Layered Chemical Identifier) program (version 20141028).^65^ Hit selection criteria were aggregated for duplicate structures using LyChi-3 as provided by the NCATS Resolver. This was all done within the Palantir Technologies Foundry Platform (Washington, DC), which is configured to ingest all HTS results generated at NCATS and harmonized these data with other sources such as ChEMBL and OrthoMCL. All qHTS screening results are publicly available at PubChem (AIDs 1508617, 1508618, 1508619, 1508620).

### Gel-Based Binding Assay

The Gel-based binding assay was performed by incubating 1 μM GST-CIB1 with the compound in dose–response (60 μM, 1:2, 4-points, *n* = 1) for 1 h at RT. After incubation, 400 nM of F-α_IIb_ was added to each tube and incubated for a further 15 min. To each reaction, 5× loading buffer (50 mM Tris–HCl, pH6.8, 10% glycerol, 0.005% bromophenol blue) was added and samples were resolved using 10% SDS-PAGE. The level of F-α_IIb_ peptide in complex with GST-CIB1 was measured by taking fluorescence images of gels using ChemiDoc MP Imaging System (Bio-Rad).

### Intrinsic Tryptophan Fluorescence (ITF) Assay

ITF was performed using a Tecan Infinite 200 Pro Fluorescence spectrophotometer as described previously.^16,33^ Ten μM GST-CIB1 in assay buffer (10 mM HEPES, pH 7.4, 125 mM NaCl, 5 mM CaCl_2_, and 0.25 mM DTT) was incubated with 10 μM unlabeled α_IIb_ peptide for 15 min, and the fluorescence was measured with an excitation wavelength 295 nm and emission at 345 nm. To assess the effect of compounds identified in our screen, 10 μM of GST-CIB1 was incubated with various concentrations of compounds at RT for 1 h before incubating with the unlabeled peptide.

### Microscale Thermophoresis (MST) Protein and Small-Molecule Binding

The binding affinity of the compounds to the CIB1-(His)_6_ protein was evaluated using microscale thermophoresis (MST). The recombinant protein was labeled with a fluorophore using Monolith His-tag labeling RED-tris-NTA second Generation kit (Nanotemper Technologies, Munich, Germany) following the manufacturer’s protocol. Compounds were titrated in a 2-fold dilution series (16 points, final concentration range of 6.1 nM to 200 μM) and incubated with the same volume of 50 nM (final concentration; 25 nM RED-tris-NTA) labeled recombinant protein for 30 min at RT (20 μL final assay volume). Measurements (in triplicate) were carried out in assay buffer (5 mM HEPES, pH 7.4, 125 mM NaCl, 5 mM CaCl_2_, and 0.01% Tween20) and standard capillaries using a Monolith NT.115 instrument (Nanotemper Technologies, Munich, Germany) with 50% LED excitation power, 40% MST power, MST on-time of 30 s, and off-time of 5 s. The dissociation constant (*K*_d_) values were calculated by fitting the thermophoresis signal at 20 s of the thermograph using the MO Affinity Analysis software (Nanotemper Technologies, Munich, Germany) and confirmed using GraphPad Prism.

For the counter screen, hexa-histidine peptides were labeled the same way as described above. Fifty nM labeled peptides were incubated with compounds at different concentrations, and the MST traces were collected as described above.

### MST Competitive Binding Study

The binding affinity of the compounds to the CIB1-(His)_6_ protein in the presence of α_IIb_ peptide was evaluated using FITC-labeled α_IIb_ as well. Following manufacturer’s recommendation,^66^ we first determined a minimum concentration of F-α_IIb_ by performing a titration of the fluorophore (1 μM, 1:2, 8-points; final concentration range of 7.8 nM to 1 μM) in a 10 μL assay volume. Solutions were placed in standard capillaries and evaluated for the fluorescent signal using the Monolith NT.115 instrument. With a desired RFU of >200 at an LED power of 50%, we selected 200 nM to determine the *K*_d_ of the protein. CIB1-(His)_6_ protein was titrated in a 2-fold dilution series (16-points, final concentration range of 15 nM to 500 μM), at a final volume of 12.5 μL. An equal volume of [2×] 200 nM FITC-labeled peptide was then added to each well and mixed, for a total volume of 25 μL. Solutions were placed in standard capillaries, incubated for approximately 1 h, and evaluated for a fluorescent signal with 50% LED excitation power, 20, 40, or 60% MST power (low, medium, high, respectively), MST on-time of 30 s, and off-time of 5 s. Based on best fit parameters in the MO Affinity Analysis software, we moved forward with medium MST power and calculated a *K*_d_ of 2.6 μM. Again, following the manufacturers recommendation, we used a factor of (1.5 × *K*_d_) and moved forward with a protein concentration of 4 μM protein.

Compounds were titrated in a 2-fold dilution series (16 points, final concentration range of 6.1 nM to 200 μM) and mixed with an equal volume CIB1-(His)_6_ protein and F-α_IIb_ peptide (final concentrations of 4 μM and 200 nM, respectively). Solutions were incubated for 1 h at RT, then placed in standard capillaries, and evaluated for fluorescent signal (in triplicate) at the following parameters: 50% LED excitation power, 40% MST power, MST on-time of 30 s, and off-time of 5 s using Monolith NT.115 instrument. Dissociation constant (*K*_d_) values were calculated by fitting the thermophoresis signal at 20 s of the thermograph using the MO Affinity Analysis software (Nanotemper Technologies, Munich, Germany) and confirmed using Graphpad Prism 7.

### *In Silico* Analysis

To gain deeper insights into the relationship between the activity of the compounds (NCGC00071855, NCGC00351170, and NCGC00351154) and their molecular structures, we conducted molecular docking studies. The crystal structure of CIB1 (PDB ID: 1XO5)^67^ was retrieved from the Protein Data Bank database and was prepared for docking procedure using Protein Preparation Wizard of the Schrödinger Suite (Schrödinger Release 2019–2, Schrödinger, LLC, New York, NY, 2019). During the protein preparation, hydrogen atoms were added, water molecules were removed, and optimal protonation states and ASN/GLN/HIS flips were determined. Residues Leu131, Ile153, and Phe173 were found to be reported to play a key role in ligand binding.^12,18,33,48,59,67^ Thus, the active site was defined as encompassing a 20 Å around these residues (*X*: 33.56, *Y*: −4.96, *Z*: 6.89) (Figure S11). The LigPrep module of the Schrodinger Suite was used to generate the correct protonation states for the ligands, which were then used for the docking studies. The OPLS3e force field^68^ was applied for the minimization of the structures, and different ionization states were generated by adding or removing protons from the ligand at a target pH of 7.0 ± 2.0 using Epik version 3.1.^49,69^ Tautomers were also generated for each ligand. To generate stereoisomers, the information on chirality from the input file for each ligand was retained, as is for the entire calculation. Docking was performed using the GlideXP scoring function implemented in Maestro.^50,70^

### Platelet Preparation and Aggregation

Whole blood was drawn by venipuncture from healthy adult volunteers (of both gender and race) with informed consent. Approval was obtained from the institutional review boards of Thomas Jefferson University, according to the Declaration of Helsinki. Blood was collected in acidified citrate dextrose as an anticoagulant. Platelet-rich plasma (PRP) and washed platelets were prepared, as previously described.^44^ Platelet aggregation was performed using washed platelet suspensions containing 2 × 10^8^ platelets/mL using a Chrono-Log Lumi-Aggregometer (Chrono-Log), as described previously.^11^ Aggregation traces were recorded by using Aggrolink software (Chrono-Log). Thrombin-induced P-selectin (CD62P) exposure was performed using Accuri C6 flowcytometer (BD Bioscience) as described previously.^11^

### Statistical Analysis

Statistical analysis of the data was performed using Student’s *t* test (mean ± standard error of the mean). *P* ≤ 0.05 was regarded as statistically significant. Each experiment was repeated independently at least 3 times.

## Supporting Information Available

Assay optimization; curve class definitions; gel performance; thiol reactivity dose-response curves; microscale thermophoresis assay optimization; platelet aggregation curves; representative gel of protein purity; and binding example (PDF)
Assay protocol; primary screen in 1536-well format; confirmatory screen; 384-well confirmatory and counter screen; quantified gel results; thrombin assay results; and analog details (XLSX)
