# Supplementary material for: Small-Molecule Disruptors of the Interaction between Calcium- and Integrin-Binding Protein 1 and Integrin αIIbβ3 as Novel Antiplatelet Agents
Source: ACS Pharmacol Transl Sci. 2024 May 29;7(7):1971–82. doi: 10.1021/acsptsci.4c00026 (PMC11249646; doi:10.1021/acsptsci.4c00026)
Supplement: Supplementary file 1 — pt4c00026_si_001.pdf [file pt4c00026_si_001.pdf]

Small molecule disruptors of the interaction between calcium- and integrin-binding protein 1 and integrin $\alpha_{IIb}\beta_3$ as novel anti-platelet agents

Kalyan Golla[1†] Adam Yasgar[2†], Voddarahally N. Manjuprasanna[1], Meghna U. Naik[1], Bolormaa Baljinnyam[2], Alexey V. Zakharov[2], Sankalp Jain[2], Ganesha Rai[2], Ajit Jadhav[2], Anton Simeonov[2*], and Ulhas P. Naik[1*]

[1]Cardeza Center for Hemostasis, Thrombosis, and Vascular Biology, Cardeza Foundation for Hematologic Research, Department of Medicine, Thomas Jefferson University, Philadelphia, PA, United States of America.

[2]National Center for Advancing Translational Sciences, National Institutes of Health, Rockville, MD, United States of America.

*Corresponding authors: ulhas.naik@jefferson.edu; anton.simeonov@nih.gov

[†]Co-first authors

Supplemental figure legends

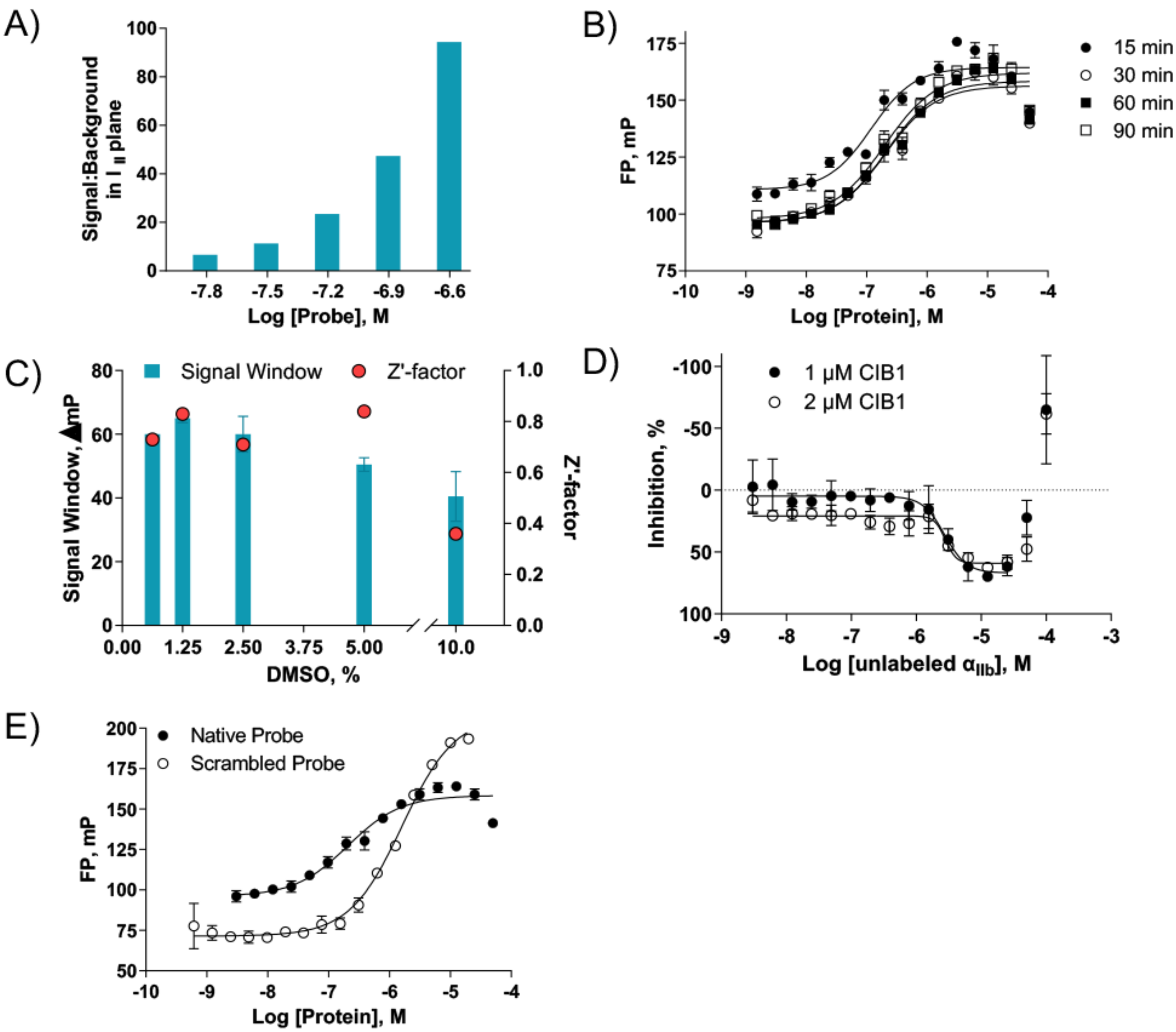

Figure S1. Assay optimization in 384-well plate. A) Concentration of 50 nM F-α_{IIb} was chosen due to it exhibited a strong S:B. B) CIB1 K_d determination and time-course. C) Percent DMSO tolerance determination. D) Unlabeled α_{IIb} peptide in 1 or 2 μM CIB1 with 50 nM F-α_{IIb} in 384-well format incubation at RT for 1 hour. E) CIB1 K_d determination with scrambled peptide (F-scrambled).

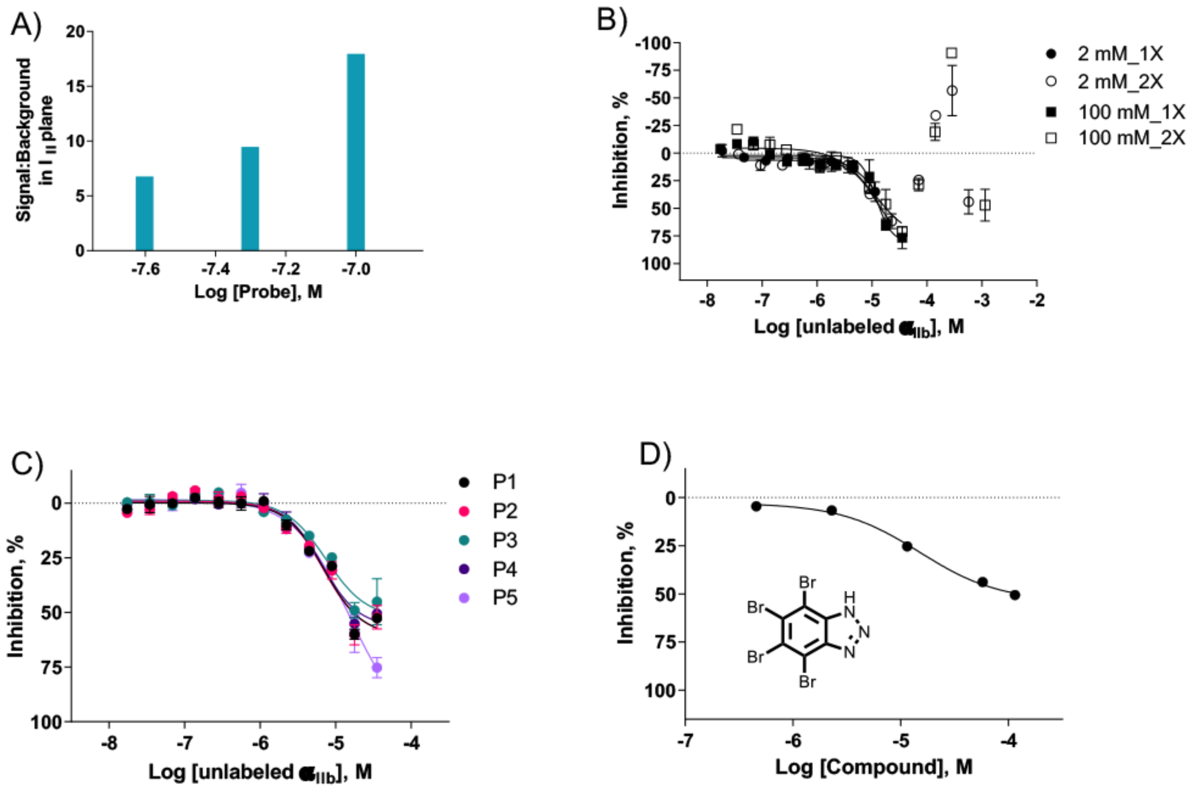

Figure S2. Assay optimization in 1536-well plates. A) Optimum concentration of F-α_{IIb} (probe) determination. B) Specific binding of F-α_{IIb} peptide (IC_{50}) determination using unlabeled peptide. C) LOPAC[1280] Screen: assay performance of intraplate control unlabeled αIIb peptide titration in 0.85 µM GST-CIB1 with 100 nM F-α_{IIb} in 1536-well format incubation at RT for 15 minutes. Mean IC_{50} of 8.6 µM and Minimum Significant Ratio (MSR) = 2.7 D) TBB (NCGC00092352) identified as technical control with an IC_{50} value of 14.1 µM.

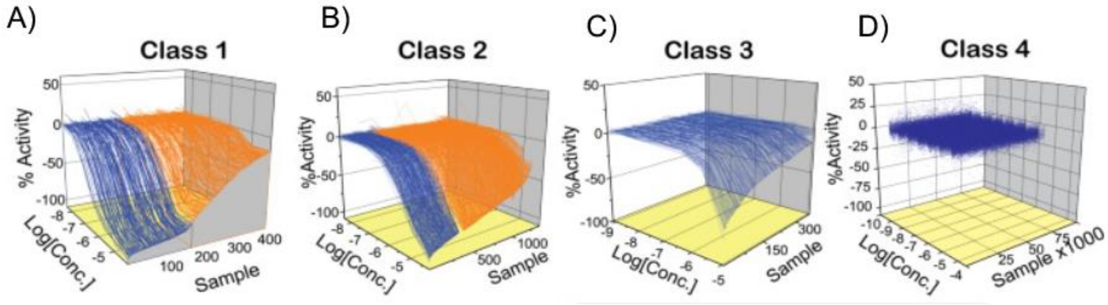

Curve Class	Description
-1.1	Full efficacy, complete curve
-1.2	Partial efficacy, complete curve
-1.3	Full efficacy, complete curve with poor fit
-1.4	Partial efficacy, complete curve with poor fit
-2.1	Full efficacy, partial curve
-2.2	Partial efficacy, partial curve
-2.3	Full efficacy, partial curve with poor fit
-2.4	Partial efficacy, partial curve with poor fit
-3	Single point activity
4	Inactive

Adapted from https://academic.oup.com/toxsci/article/167/1/282/5106019#supplementary-data

Figure S3. Example curves highlighting qHTS curve classification criteria. Lines connecting titration data corresponding to inhibitory compounds are shown. A) Classes 1.1 (blue; >80% efficacy) and 1.2 (orange; ≤80% efficacy) inhibitors display full and partial activity, respectively, with r^2 ≥0.9. B) Incomplete curves for inhibitors having AC_{50} values within and beyond the tested titration range are classes 2.1 (blue; >80% efficacy, r^2 >0.9) and 2.2 (orange; ≤80% efficacy, r^2 <0.9), respectively. C) Incomplete inhibitory (blue) curves that show weak activity and poor fits are class 3. D) Inactive compounds are Class 4.

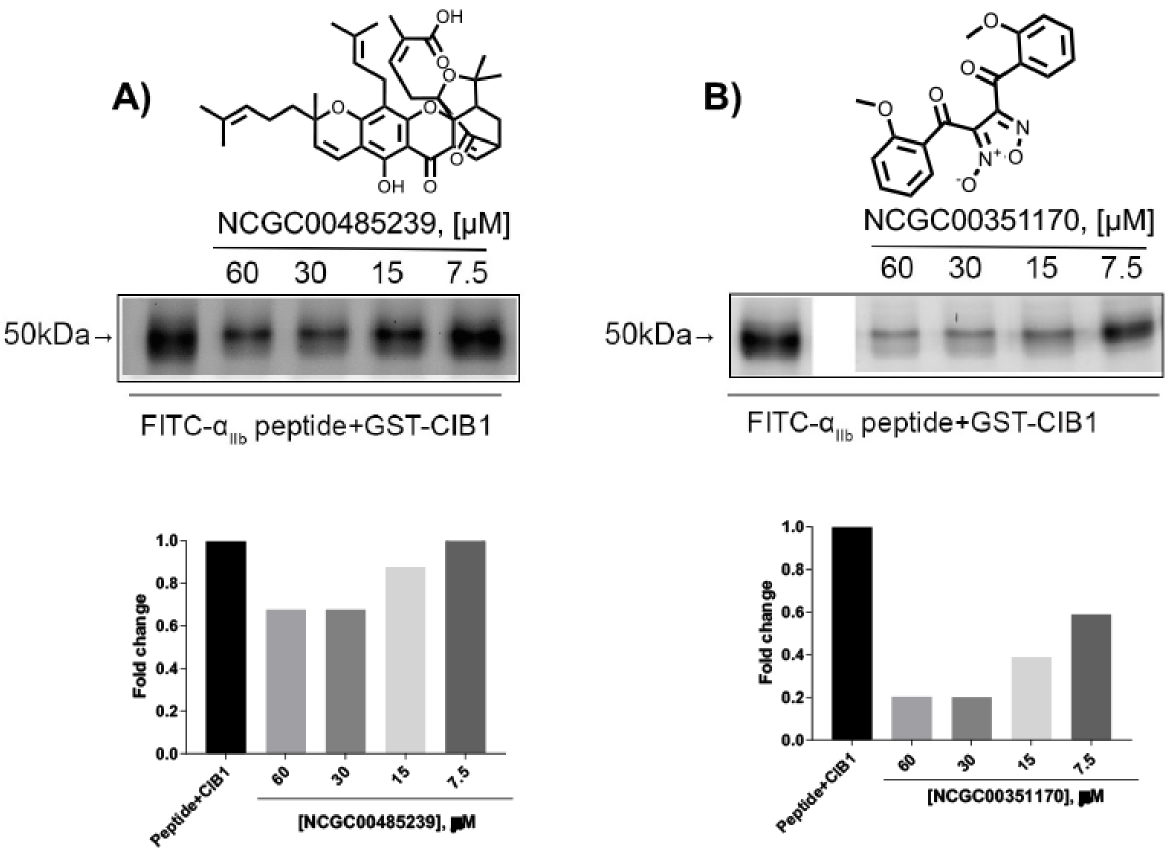

Figure S4. In-gel Assay. Compounds at indicated concentrations were pre incubated with 1 μM GST-CIB1 for 1 hour. After incubation, 400 nM of F-αIIb peptide was added to each reaction and incubated for 15 minutes. Samples were resolved using SDS-PAGE gels and bound $F_{\text{-αIIb}}$ peptide visualized by fluorescent Bio-Rad gel imager. An example gel of a weak active (low inhibition) and active (exhibiting inhibition) compounds are shown in panel A and B, respectively.

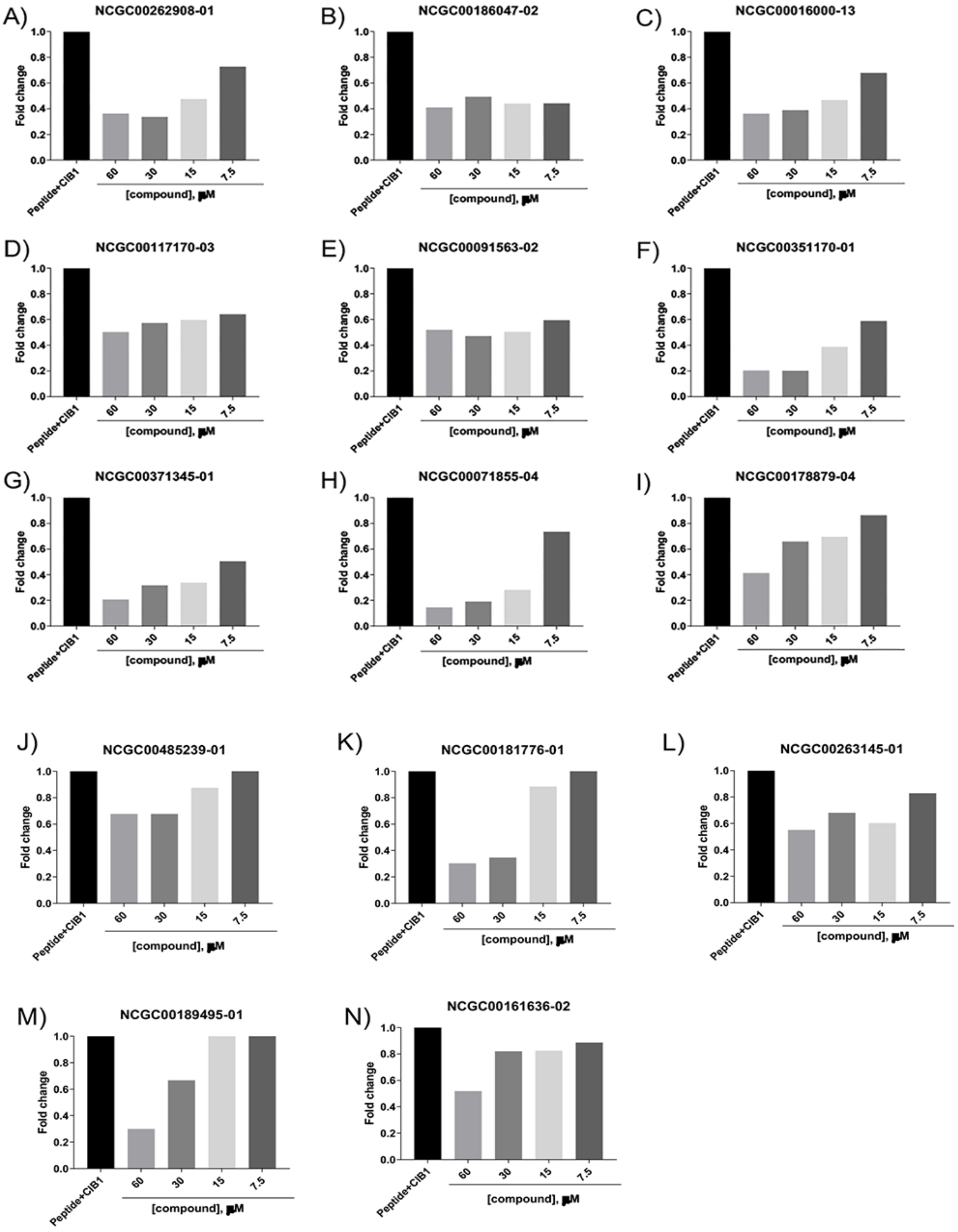

Figure S5. In-gel Assay quantification of candidate inhibitors.

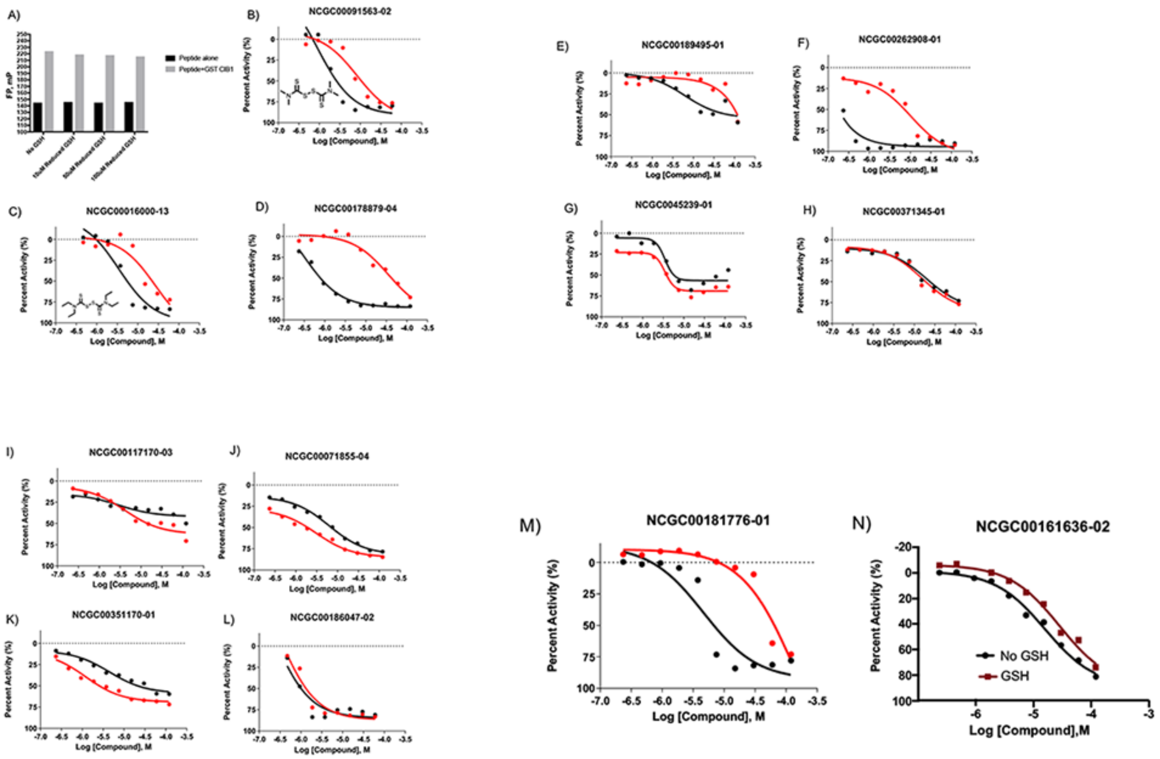

Figure S6. Effect of GSH on selected compounds. A) Effect of GSH on F-αIIb peptide binding to GST-CIB1. Various concentrations of reduced GSH incubated with 1 µM of GST-CIB1 for 30 minutes. After incubation, 100 nM of F-α_{IIb} added to each condition and further incubated for 15 minutes. Amount of peptide binding to protein was measured by fluorescence polarization.

B to J) Various doses (from 0.23 to 120 µM) of selected compounds were incubated with 1 µM GST-CIB1 for 1 hour in the presence (●) or absence (●) of 10 µM reduced GSH followed by incubation with 100 nM F-α_{IIb} peptide for 15 minutes, followed by FP measurement.

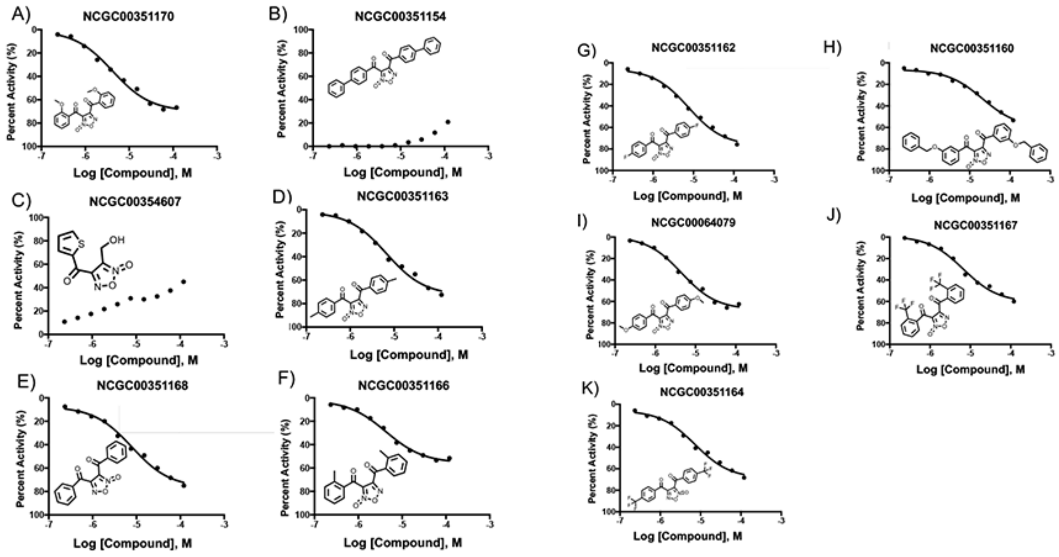

Figure S7. FP assay performance of NCGC00071855 and NCGC00351170 analogs with IC_{50} values ranging from 4.2 to 20.7 μM.

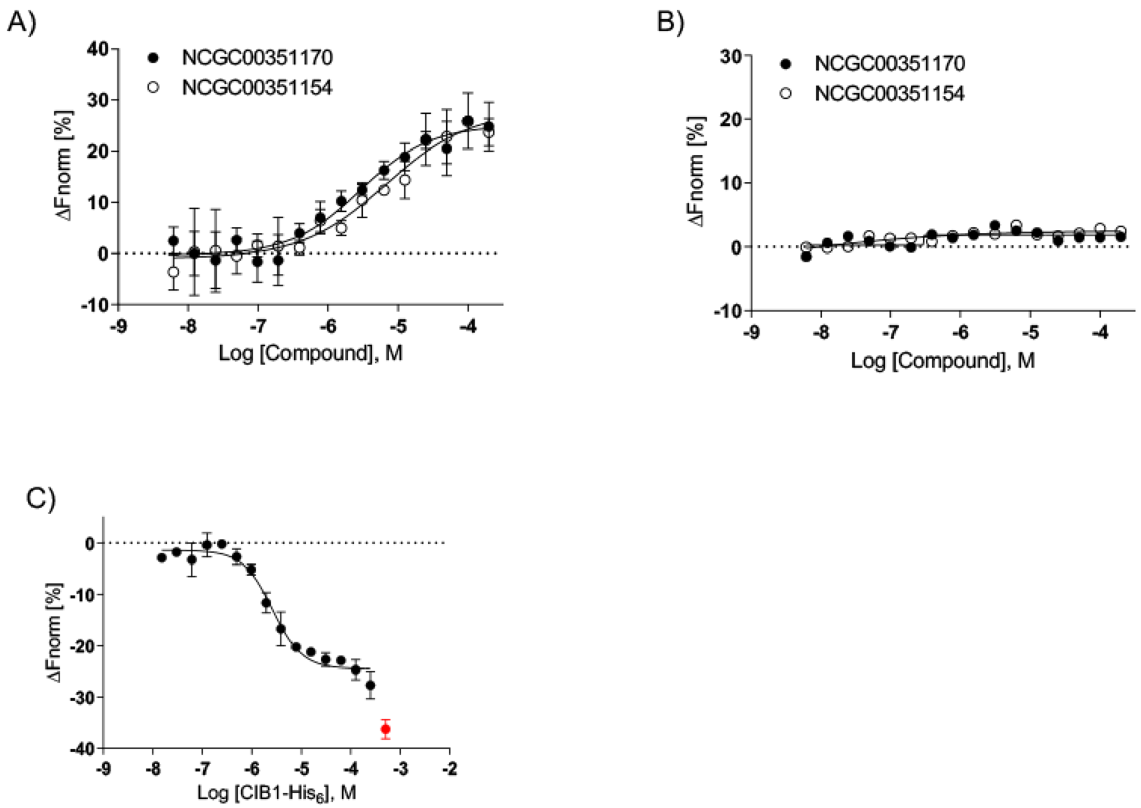

Figure S8: Panel (A) Both compounds exhibited binding to CIB1, with equilibrium dissociation constants (K_d) of 2.9 and 6.7 μM, respectively. As shown in (B), the compounds do not interference with the histidine tag on CIB1 and/or the fluorophore used for the MST assay. Panel (C) K_d of 2.6 μM measured for the binding of F-α_{IIb} to the same CIB1-(His)$_6$ protein.

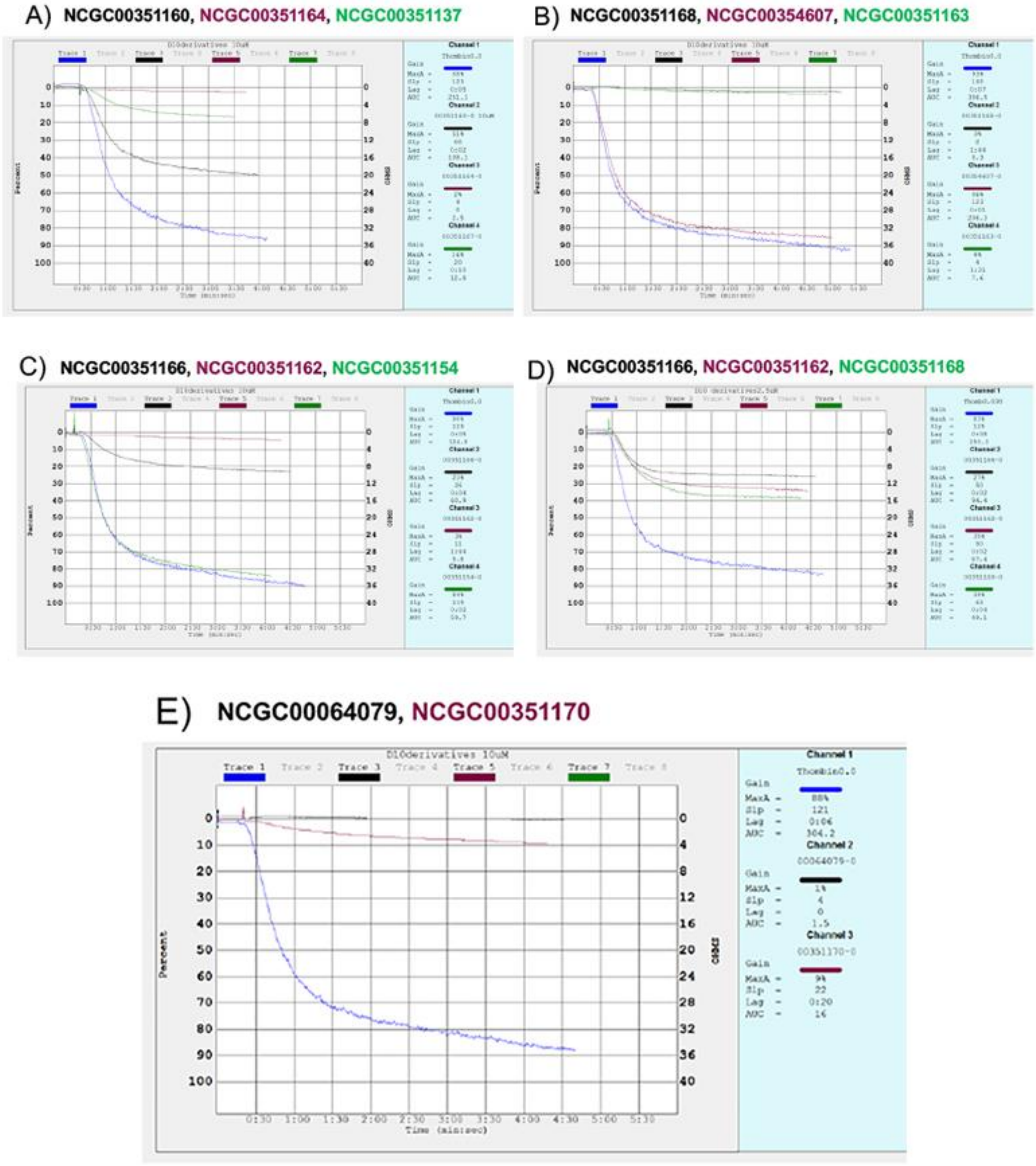

Figure S9: Effect of compounds on human platelet activation. Representative platelet aggregation tracings of washed human platelet suspensions treated with vehicle (DMSO) or indicated compounds at 10 μM for 30 minutes at 37°C followed by activation with thrombin (0.03U/mL).

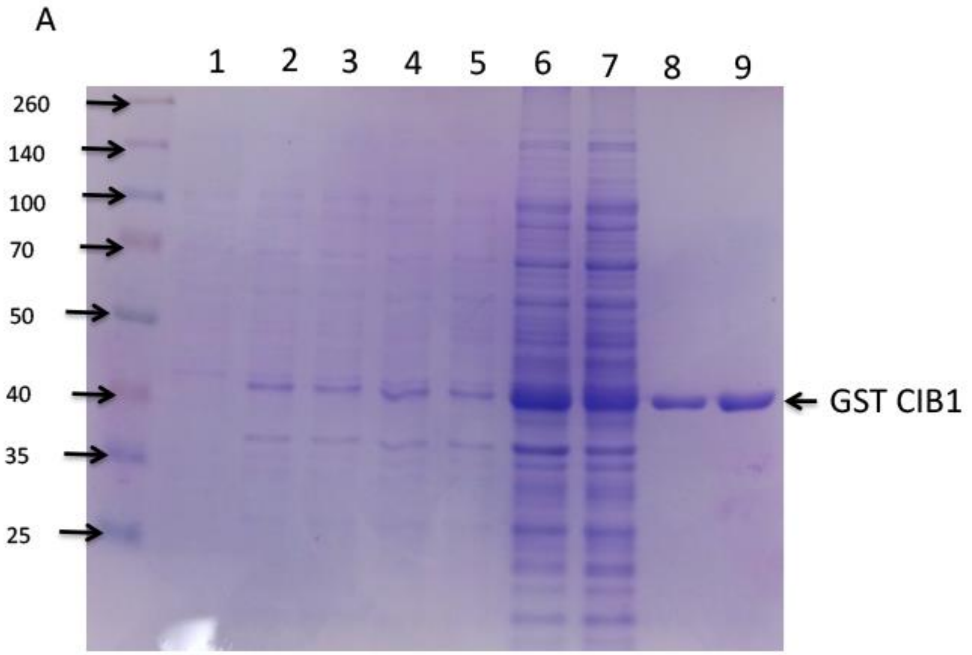

1. No induction
2. 2hrs after induction (3,4,5)
6. Lysate
7. Supernatant of lysate

8. Elute
9. 1:20 Dilution of protein (2.05 µg)

Figure S10: GST-CIB1 protein production. Representative gel and visualization of GST-CIB1 used for testing.

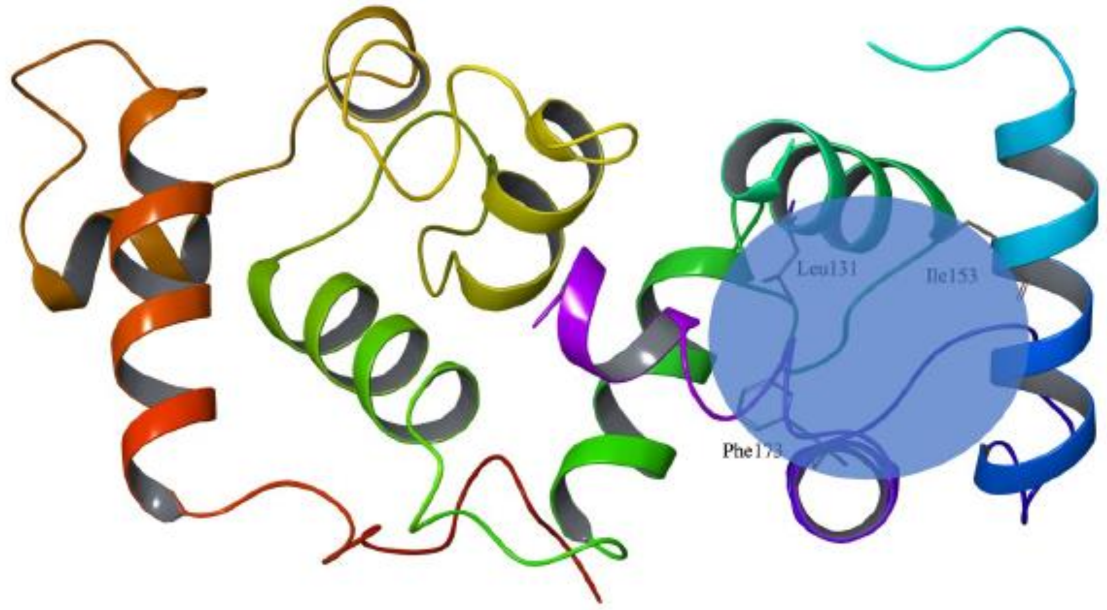

Figure S11: Structure of CIB1 (PDB ID: 1XO5). The blue highlighted part depicts the protein binding pocket.
